# Use of stable isotopes to reveal trophic relationships and transmission of a food-borne pathogen

**DOI:** 10.1038/s41598-024-53369-6

**Published:** 2024-02-02

**Authors:** Émilie Bouchard, Michaël Bonin, Rajnish Sharma, Adrián Hernández-Ortiz, Géraldine-G. Gouin, Audrey Simon, Patrick Leighton, Emily Jenkins

**Affiliations:** 1 Department of Veterinary Microbiology, Western College of Veterinary Medicine, University of Saskatchewan, 52 Campus Drive, Saskatoon, SK S7N 5B4 Canada; 2 Research Group on Epidemiology of Zoonoses and Public Health (GREZOSP), Faculty of Veterinary Medicine, Université de Montréal, Saint-Hyacinthe, QC J2S 2M2 Canada; 3 Département de Biologie, Centre d’études nordiques, Université Laval, Quebec, QC G1V 0A6 Canada; 4 Nunavik Research Centre, Makivvik Corporation, Kuujjuaq, QC J0M 1C0 Canada; 5 Centre de recherche en santé publique de l’Université de Montréal et du CIUSSS du Centre-Sud-de-l’Île-de-Montréal, Montreal, QC H2L 2W5 Canada

**Keywords:** Parasitology, Microbiology, Ecology, Stable isotope analysis

## Abstract

Predators in food webs are valuable sentinel species for zoonotic and multi-host pathogens such as *Toxoplasma gondii*. This protozoan parasite is ubiquitous in warm-blooded vertebrates, and can have serious adverse effects in immunocompromised hosts and foetuses. In northern ecosystems, *T. gondii* is disproportionately prevalent in Inuit people and wildlife, in part due to multiple routes of transmission. We combined data on *T. gondii* infection in foxes from Nunavik (northern Québec, Canada) with stable isotope data tracking trophic relationships between foxes and several of their main prey species. Red (*Vulpes vulpes*) and Arctic fox (*Vulpes lagopus*) carcasses were collected by local trappers from 2015 to 2019. We used magnetic capture PCR to detect DNA of *T. gondii* in heart and brain tissues, and enzyme-linked immunosorbent assay to detect antibodies in blood. By linking infection status with diet composition, we showed that infected foxes had a higher probability of consuming aquatic prey and migratory geese, suggesting that these may be important sources of *T. gondii* transmission in the Arctic. This use of stable isotopes to reveal parasite transmission pathways can be applied more broadly to other foodborne pathogens, and provides evidence to assess and mitigate potential human and animal health risks associated with *T. gondii* in northern ecosystems.

## Introduction

**Figure 1:**
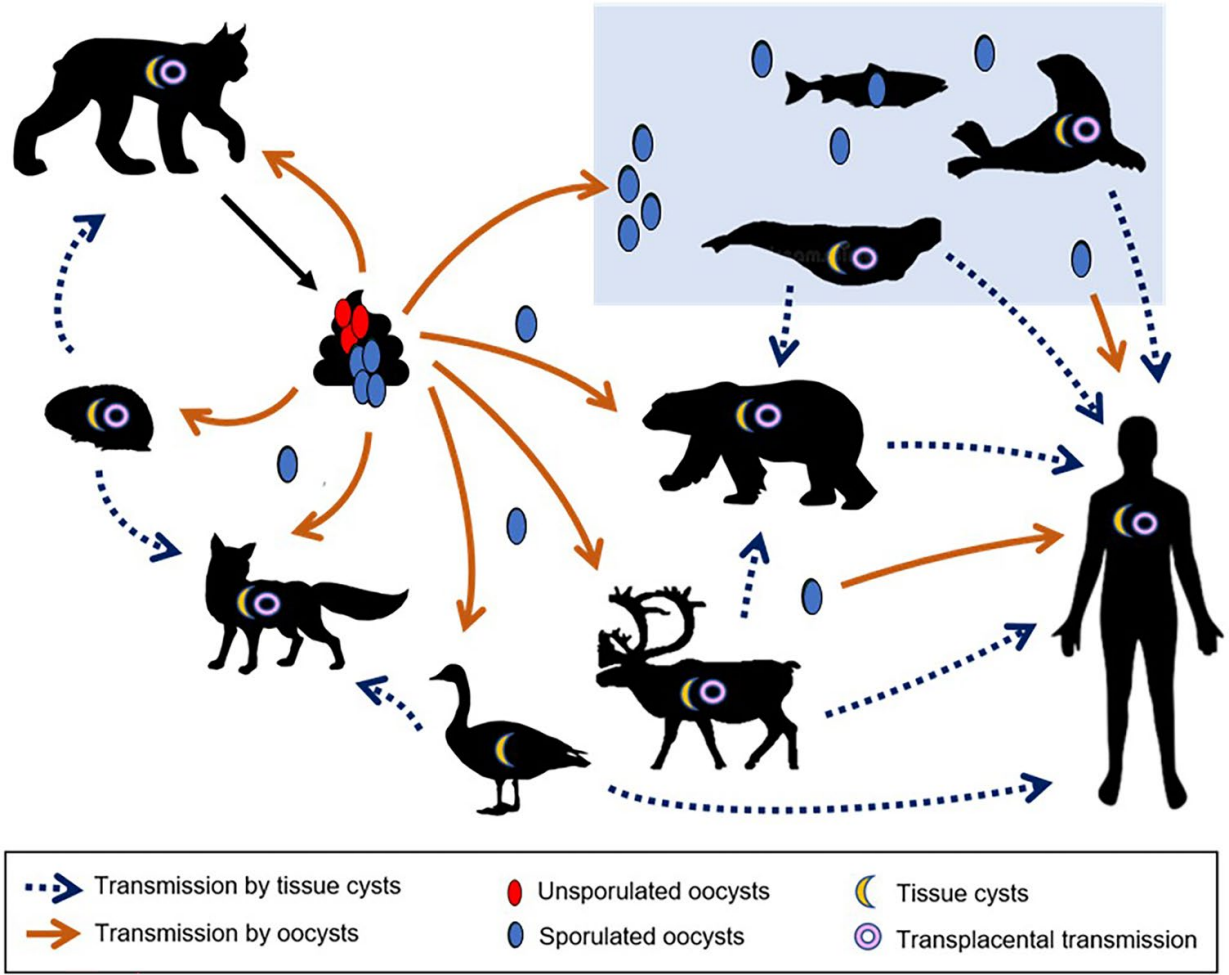
Potential routes of transmission of the zoonotic parasite *Toxoplasma gondii* in the North, with focus on free-ranging wildlife hosts and the shared environment (Reprinted and modified with permission from Springer Nature: Springer, Toxoplasmosis in Northern Regions, Bouchard et al.^12^).

The protozoan *Toxoplasma gondii* is a ubiquitous, zoonotic parasite of public health significance. In humans, *T. gondii* can cause life-threatening infections, especially in immunocompromised hosts and foetuses^1,2^. Exposure to the parasite in some Inuit communities in Nunavik, northern Québec, Canada, is much higher than in other parts of North America (43% seroprevalence compared to 10–15%)^3^. Inuit are thought to be primarily exposed through consumption of terrestrial and aquatic Arctic wildlife, often prepared in ways that would not inactivate the parasite^3,4^. Still, transmission of *T. gondii* in terrestrial Arctic ecosystems is complex, potentially involving food, water and vertical routes, and food-borne source attribution is often unclear. Northern transmission of *T. gondii* could involve food or water contaminated with oocysts^3,5^; oocysts of *T. gondii* have been observed in aquatic filter feeders (i.e., fish, oyster, clam, snail)^6,7^. However, true food-borne transmission from ingesting tissue cysts of the parasite in infected intermediate hosts (versus exposure to oocysts) is thought to be significant in the Arctic, as wild and domestic felids, the only known source of oocysts in the environment, are mostly absent in tundra and high Arctic ecosystems^8^. Therefore, northern hosts may be exposed through the consumption of cysts of *T. gondii* in the tissues of chronically infected migratory prey, such as caribou (*Rangifer tarandus*), Arctic-nesting geese (e.g. *Branta canadensis, Anser caerulescens)* or marine mammals^5,9–11^ (Fig. 1).

Given their generalist predator and/or scavenger foraging behaviors across multiple trophic levels, and their close association with human activities, foxes (*Vulpes* spp.) are potentially good sentinel species to investigate *T. gondii* transmission through food webs. Indeed, exposure to *T. gondii* (i.e., seroprevalence) in foxes tracks closely with that of people in many regions of the Canadian North^13^. However, documenting trophic routes through which people and foxes in remote regions are infected with *T. gondii* remains challenging. Stable isotope analysis, an indirect diet reconstruction approach, has been used to study the diet of terrestrial species, including foxes^14–18^. Carbon (δ^13^C) and nitrogen (δ^15^N) isotopic values of consumer tissues reflect dietary composition, after accounting for trophic enrichment against heavier isotopes and assimilation^19,20^. Carbon isotopic ratios distinguish between terrestrial and marine food sources, while nitrogen isotopic ratios are a relative index of trophic position of individuals or species^21,22^. Different body parts provide dietary information that is integrated over different time scales (e.g., days to months), due to the turnover rate of isotopes^19,20,23^. For example, isotopic ratios of blood cells and muscle tissue provide insights on the food habits of a consumer over the past few weeks, while hair reflects the diet of the animal during the entire growth period of the hair since the last molt^23^.

In this study, we combined data on stable isotope analysis and pathogen infection status to determine possible routes of transmission of *T. gondii* in a highly affected region of the Canadian Arctic^3^. It has been suggested that *T. gondii* may enter the terrestrial Arctic ecosystem of northern Norway via migratory birds^24^; an estimated 7% of barnacle geese (*Branta leucopsis*) on Svalbard, where no wild or domestic felids are present, are exposed to the parasite^5^. Previous work on migratory geese and marine mammals in Nunavik found a seroprevalence of 11% in migratory geese (primarily *Branta canadensis*), and 20% in ringed seals (*Pusa hispida*)^25^, and recent findings in the 2017 Nunavik Inuit Health Survey reported higher seroprevalence for *T. gondii* in Inuit with consumption of marine mammals (especially seal), fish, and geese^3^. We therefore hypothesize that migratory geese and aquatic hosts consumed by foxes may be primarily responsible for transporting *T. gondii* to the Arctic and disseminating the parasite within northern ecosystems. Given the zoonotic risk posed by *T. gondii* in Inuit communities, documenting its prevalence and transmission routes in northern wildlife is crucial to understanding and addressing the risk of infection in vulnerable animal and human populations. More broadly, we demonstrate that linking epidemiological and ecological approaches provides a powerful method to unravel transmission of *T. gondii* and other food-borne pathogens with complex transmission routes.

## Methods

### Study area

**Figure 2:**
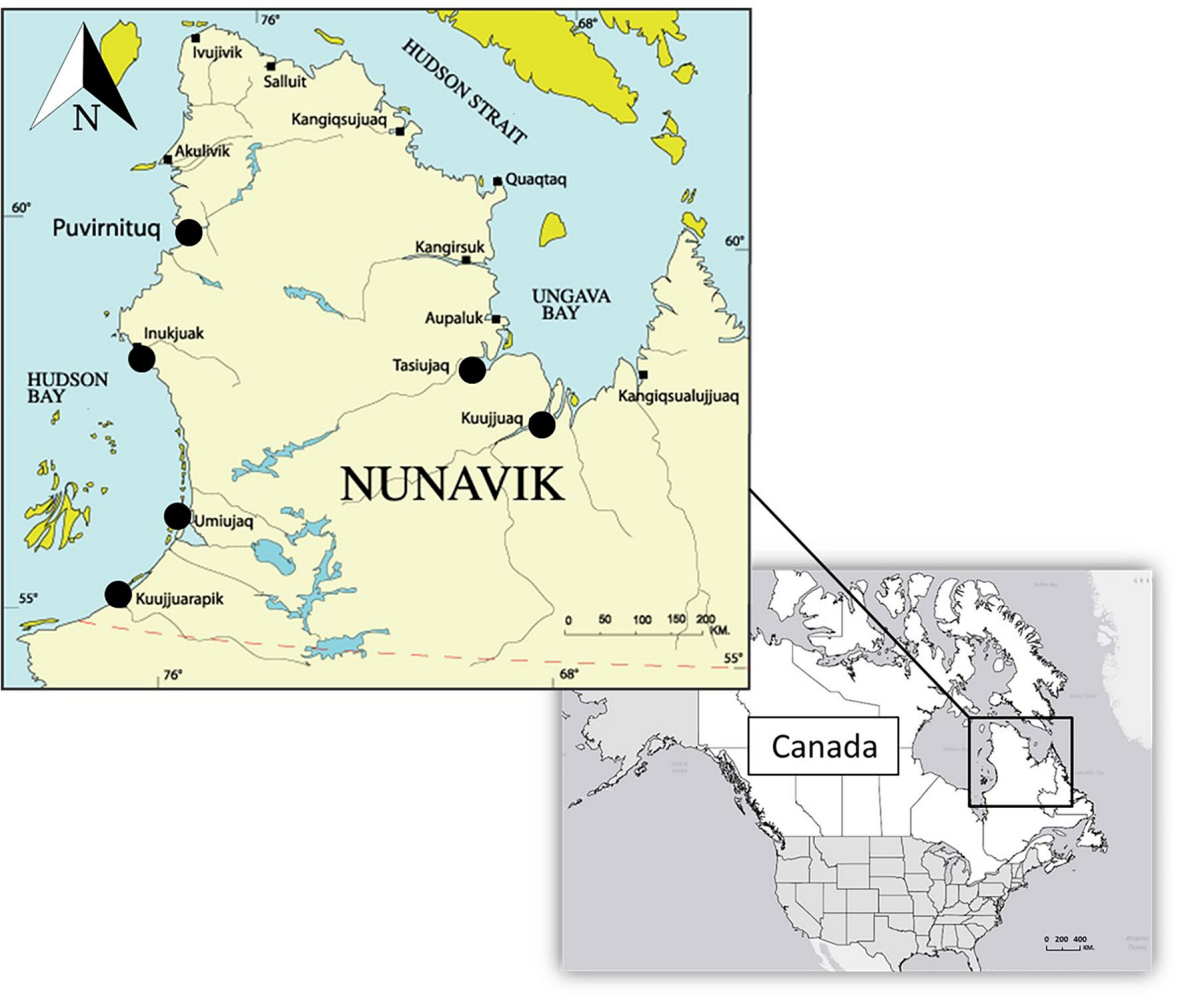
Communities (black circles) and surrounding areas where foxes (*Vulpes* spp.) were collected during winter 2015–2019 by trappers from Nunavik, Canada (Reprinted and modified with permission from Makivvik Corporation (https://www.makivvik.ca/wp-content/uploads/2013/02/nunavik1.gif).

The climate in Nunavik, northern Québec (Canada), is heavily influenced by two large water bodies, Hudson Bay and Ungava Bay (Fig. 2), with ice covering the sea from November to July. The northern part of Nunavik has an Arctic climate, continuous permafrost, tundra vegetation, and an annual average temperature of − 7.5 °C and precipitation of 300 mm. The southern, inland part of Nunavik has a Subarctic climate, discontinuous permafrost, taiga vegetation and an average annual temperature of − 2.5 °C and precipitation of 700 mm^26^. Potential terrestrial food sources (through predation and scavenging) for Arctic and red foxes include lemmings (*Lemmus* spp.), voles (*Microtus* spp.), Arctic hares (*Lepus arcticus*), Arctic ground squirrels (*Urocitellus parryii*), migratory woodland caribou (*Rangifer tarandus caribou*), muskoxen (*Ovibos moschatus*), and Arctic nesting geese (e.g. *Branta canadensis*, *Anser caerulescens*)^27,28^. Both fox species rely heavily on lemmings and voles^29^. Foxes may also scavenge carcasses of marine wildlife^30,31^, including beluga (*Delphinapterus leucas*), walrus (*Odobenus rosmarus*), seals *(Phoca hispida, Erignathus barbatus),* and fish, as natural mortalities or harvested by human residents^32^.

### Fox sampling

This study was part of a larger research project on *T. gondii* in foxes across northern Canada^13^. Fox carcasses were harvested by local trappers during regular, licensed fur-trapping activities during winters of 2015–2019 (between December and February) in two regions of Nunavik: eastern Hudson Bay (Arctic foxes = 23, red foxes = 51) and southwestern Ungava Bay (Arctic foxes = 19, red foxes = 146) (Fig. 2). Following consultation, interested communities self-elected for participation, and local coordinators were recruited to ensure that carcasses were stored at − 20 °C until shipped for necropsy.

Frozen carcasses were sent for necropsies at the Western College of Veterinary Medicine in Saskatoon (SK), the Faculté de Médecine Vétérinaire in Saint-Hyacinthe (QC), and the Nunavik Research Centre in Kuujjuaq (QC). Acknowledging that trappers may have superficially cross-contaminated fox carcasses while skinning, we sampled deep muscle tissue and internal organs using clean tools between carcasses. From each fox, we collected whole hearts, brains, thigh muscle, and hair (extracted by tweezers from root to tip from residual hair patches left on the right hind paw by trappers). Brain and heart tissues are predilection sites for *T. gondii* in animals^33–35^. We recorded harvest location, species, and sex for all individuals. In accordance with the Canadian Council on Animal Care guidelines, this research was exempt from Animal Research Ethic Board review because all samples were collected from animals legally harvested for non-research purposes in accordance with relevant guidelines and regulations.

### Serological analysis

As validated in Sharma et al.^36^ and described in Bouchard et al.^13^, we detected antibodies to *T. gondii* in fluid from thawed heart tissue (diluted 1:2) using the commercially available ID Screen® Toxoplasmosis Indirect Multi-species Enzyme-linked immunosorbent assay (ELISA) (IDvet, Grabels, France). Samples with an S/P % less than or equal to 40% were considered negative. Samples greater than or equal to 70% were considered positive. If the S/P % was between 40 and 70%, the test result was considered doubtful, and testing was repeated. The ELISA relative sensitivity and specificity using magnetic capture PCR as a reference test was 94% and 100%, respectively^37^.

### Molecular analyses

As validated by Opsteegh et al.^38^ and described in Bouchard et al.^13^, sequence specific DNA of *T. gondii* was extracted by magnetic capture from whole heart and brain tissues combined for each fox followed by real-time PCR using the Tox 9F (5′-aggagagata tcaggactgtag-3′) and Tox 11R (5′-gcgtcgtctc gtctagatcg-3′) primers, for the detection of the 188 bp *T. gondii* sequence within the 529 repeat-element. Each run on magnetic capture included two spiked beef samples (positive controls) and one beef sample without spiking (negative control). A reaction was considered positive if (1) the Cq value was less than or equal to 35, (2) the two positive extraction controls were positive, and (3) the negative and two no-template controls were negative. Reactions with Cq values between 35 and 40 were considered positive if a 188 bp band was identified on gel electrophoresis.

### Collection of food sources

Potential major food sources of foxes were collected from a concurrent study in Nunavik^22^. Briefly, hair and muscle samples from prey were collected in 2016–2017 and 2017–2018 in the same geographic regions as foxes. We selected three dietary endpoints of red and Arctic fox diets^29^: hair from Ungava collared lemmings (*Dicrostonyx hudsonius*), breast muscle from Canada geese collected during their southern migration (Aug-Sept) (*Branta canadensis*), and ventral muscle of fish, both anadromous Arctic char (*Salvelinus alpinus*) and freshwater lake trout (*Salvelinus namaycush*). Geese, Arctic char, and lake trout were previously found to be infected with *T. gondii* in the region^25,39^, making them plausible sources of exposure. For logistical reasons, we did not attempt to fully describe all potential dietary sources of foxes, but rather to focus on distinct isotopic endpoints that represent different sources of *T. gondii* introduction (resident terrestrial vs migratory geese vs aquatic).

### Stable isotope analysis

We collected hair and muscles from foxes trapped in winter. Since foxes molt twice per year^14,40^, with the autumn molt starting in September, stable isotope signatures from winter fur in our study represented diet composition of foxes from the preceding fall (September to December). Isotopic ratios of muscle tissues from foxes in our study represented their diet composition during winter (December to February)^17^. Variations in lipid concentration can significantly influence δ^13^C measurements^41^; therefore, we cleaned fox and lemming hair with distilled water, soaked 3 times for 10 min in a 2:1 chloroform:methanol solution to remove lipids, then rinsed hair again in distilled water before drying at room temperature for 48 h. We cut multiple, complete hairs in 1 mm segments into tin cups to achieve a total weight of 1 mg from each fox or lemming. We lyophilised muscle tissues (foxes and food sources) for 36 h and ground them in a ball mill. To remove lipids, we added a 2:1 chloroform:methanol solution to each muscle sample, mixed with a tube stirrer, and centrifuged for 8 min at 10,000 RPM. The supernatant was removed, and we repeated the procedure until the supernatant was clear. The samples were left to dry for 24 h, then 1 mg was weighed into tin cups^42^.

We analysed muscle and hair samples from foxes and prey for nitrogen and carbon at the Laboratoire d’Océanographie of Laval University, Québec (Canada). Isotopic analyses were performed by continuous-flow isotope ratio mass spectrometer (Thermo Electron Delta Advantage) using an ECS 4010 Elemental Analyzer/Zero Blank Autosampler (Costech Analytical Technologies). Stable isotope ratios were expressed in δ notation as parts per thousand (‰) deviation from V-Pee Dee Belemnite (carbon) and AIR (nitrogen) international standards. Measurement precision was ± 0.2‰ for δ^13^C and ± 0.1‰ for δ^15^N.

### Statistical analysis

#### Serological and molecular test agreement

Proportion of positive results was compared between ELISA and MC-qPCR, using McNemar’s Chi-square tests for paired data. The kappa coefficient (k) was used to determine the level of agreement between the two tests. Analyses were performed using IBM SPSS (ver. 26; Armonk, New York, USA).

#### Prevalence and risk factors

Seroprevalence, tissue prevalence and their 95% confidence intervals (CI) were calculated from the proportion of positive results using EpiTools epidemiological calculators^43^. We used linear regression to test for the effects of sex, species, region, and status of infection on stable isotope values of fox hair and muscles. We used a logistic regression with package lme4 v.1.1–26^44^ in R v.3.6.3^45^ to evaluate the effect of species, sex, region, terrestrial, aquatic, and migratory geese dietary sources on *T. gondii* prevalence (a fox was considered positive if it was positive on either serology or molecular testing). Foxes with missing data were not included in the regression. We tested the relevance of possible interaction terms by comparing models with Akaike information criterion (AIC), where models with ΔAIC < 2 were considered equally plausible. Models were tested against the null model to see if there was a significant amelioration (Table 1). The models considered were based on hypotheses of risk factors influencing *T. gondii* prevalence in foxes (i.e., region, diet, sex and species)^13^. We did not include year as a fixed effect in our model as it was dependent on region. We did include year as a random effect to reflect annual variation in ecological conditions that may influence the prevalence of *T. gondii* in the fox population (e.g., climate-related factors, fox density, lemming density). As tissues used to reconstruct diet incorporate dietary information over long timescales (weeks to months), this minimizes the effect of short-term diet variation that could not be captured by using year as a variable in our model.

**Table 1:** Akaike’s information criterion model selection results for hypotheses of risk factors influencing *Toxoplasma gondii* prevalence in foxes (*Vulpes* spp.) in Nunavik, QC.

Model	Models for fall diet	∆AIC	AIC weight	Log likelihood	k
1	Toxo ~ Region*Species + Species*Dietaquatic + Species*Dietmigration + (1|Year)	0.00	0.99	− 75.04	9
2	Toxo ~ Region*Dietaquatic + Region*Dietmigration + (1|Year)	9.70	0.01	− 82.13	7
3	Toxo ~ Dietaquatic + Dietmigration + (1|Year)	13.64	0.00	− 87.35	4
4	Toxo ~ Region + Sex + Species + (1|Year)	15.99	0.00	− 87.46	5
5 Null	Toxo ~ 1 + (1|Year)	18.75	0.00	− 92.00	2

	Models for winter diet				
1	Toxo ~ Region*Species + Species*Dietaquatic + Species*Dietmigration + (1|Year)	0.00	0.77	− 109.11	9
2	Toxo ~ Dietaquatic + Dietmigration + (1|Year)	2.52	0.22	− 115.71	4
3	Toxo ~ Region*Dietaquatic + Region*Dietmigration + (1|Year)	8.03	0.01	− 115.29	7
4	Toxo ~ Region + Sex + Species + (1|Year)	16.03	0.00	− 121.42	5
5 Null	Toxo ~ 1 + (1|Year)	13.68	0.00	− 123.35	2

AIC, Akaike’s information criterion. ΔAIC, change in AIC relative to top model. k, the number of model parameters.

#### Stable isotope analysis

To calculate the percentage of fox diet that was derived from selected terrestrial, migratory geese, and aquatic food sources, we used the δ^13^C values of lemmings, geese, and fish, respectively. As δ^13^C and δ^15^N values were very similar for goose muscles and goose eggs^22^ (t(2) = 0.01, p = 0.49), only goose muscles were used in the analysis. Geese are not present in Nunavik during winter, therefore goose signatures for this period likely represent food cached by foxes in summer for future use. These prey items of foxes were all isotopically distinct (electronic supplementary material, Tables S1, S2). Reassuringly, our isotopic values for lemming in Nunavik were similar to Robillard et al.^46^, and isotopic signatures of ringed seals (a marine mammal that consumes primarily marine fish) from Nunavik were very similar (1% difference for δ^13^C and δ^15^N) (University Laval, unpublished data) to those we described in char and trout.

We corrected red fox samples for isotopic discrimination by using values previously calculated in captive red foxes, i.e., hair: 2.6‰ for δ^13^C and 3.2‰ for δ^15^N ratios, muscle tissue: 1.1‰ for δ^13^C and 3.3‰ for δ^15^N ratios^47^. For Arctic foxes, we used values from Lecomte et al.^18^, i.e., hair: 2.2‰ for δ^13^C and 3.3‰ for δ^15^N ratios, muscle tissue: 0.4‰ for δ^13^C and 1.8‰ for δ^15^N ratios. We accounted for uncertainty in fractionation estimates in our mixed model analysis for both fox species using estimates from Lecomte et al.^18^ for muscle (SD of ^13^C = 0.1‰ and SD of ^15^N = 0.5‰) and for hair (SD of ^13^C = 0.4‰ and SD of ^15^N = 0.6‰). We used SIMMR (R-package SIMMR), a Bayesian stable isotope mixing model, to estimate the proportional contributions of each dietary endpoint (terrestrial, aquatic, and migratory geese) to fox diets. Each model consisted of four Markov Chain Monte Carlo of 1,000,000 iterations, tinned by 100 and with an initial discard of the first 1000 iterations. We performed all statistical analyses (prevalence, risk factors and stable isotope analyses) using R^45^.

## Results

### Agreement between ELISA and MC-qPCR

Twenty-six foxes were positive for antibodies to *T. gondii* on serology and negative for DNA of *T. gondii* in tissues, one was serologically negative but tissue positive, 66 were positive on both, and 135 were negative on both. There was a statistical difference between serological and molecular results (Χ^2^ = 21.3, *df* = 1, p < 0.001, n = 228), but substantial agreement between the two tests (k = 0.74). As a result, for subsequent data analyses, a fox was considered positive if it was positive on either serology or molecular testing.

### Detection of *T. gondii* antibodies and DNA

Antibodies to *T. gondii* were detected in 40% (CI_95%_: 34–47) of foxes using ELISA. *Toxoplasma gondii* DNA was detected in 29% (CI_95%_: 23–35) of foxes using MC-qPCR. As previously described^13^, seropositivity and tissue prevalence were higher in Hudson Bay versus Ungava Bay foxes (Table 2).

**Table 2:** Prevalence (% animals positive) of antibodies to *T. gondii* in blood and DNA of *T. gondii* in brain and heart tissue, and demographic variables in foxes (*Vulpes* spp.; n = 239) harvested in Nunavik, QC.

		Seroprevalence^a^ (CI_95%_)/N	Tissue prevalence (CI_95%_)/N
Species	Red fox	38% (32–45)/188	25% (20–32)/197
	Arctic fox	50% (35–65)/40	45% (31–60)/42
Sex	Male	41% (33–49)/144	28% (21–36)/150
	Female	39% (30–50)/84	31% (22–41)/88
Regions	Ungava Bay, QC	29% (22–36)/154	19% (14–25)/165
	Hudson Bay, QC	65% (54–75)/74	51% (40–62)/74
Total		40% (34–47)/228	29% (23–35)/239

N, number of individuals tested; CI, confidence intervals.

^a^Individuals not tested (no heart fluid) = 11.

### Risk factors for *T. gondii* exposure

#### Linear regression for δ^13^C and δ^15^N

**Figure 3:**
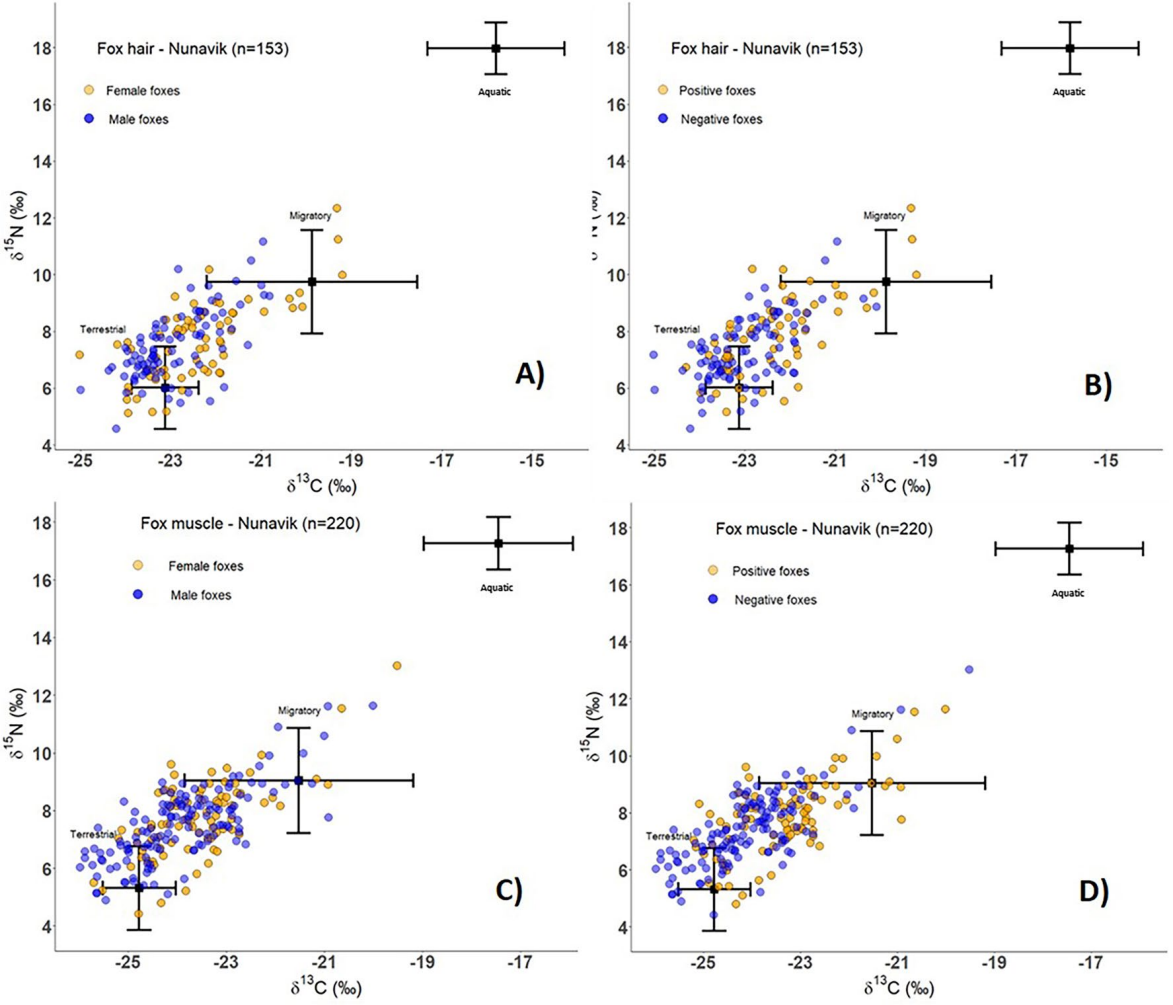
Comparisons of stable isotope (δ^13^C and δ^15^N) ratios of dietary endpoints for Nunavik foxes in fall (based on hair) and winter (based on muscle) for sex (**A** and **C**), and *Toxoplasma gondii* infection status (**B** and **D**). For both periods, female foxes consumed more aquatic food sources, and positive foxes were more likely to consume migratory geese and aquatic prey.

Male foxes had a significantly lower input of δ^13^C compared to female, and positive foxes had a significantly higher input of δ^13^C as well as δ^15^N (Table 3, electronic supplementary material Fig. S1), in both fall and winter diet. The relative contribution of the different dietary endpoints to the diet of foxes according to their infection status and sex can be seen on Fig. 3.

**Table 3:** Coefficients of the linear regression models for species, sex, region and status of infection, and effects on dietary endpoints contributions to fox diet (*Vulpes* spp.) for fall and winter seasons in Nunavik, QC.

	δ^13^C			δ^15^N		
	β (SE)	*df*	p	β (SE)	*df*	p
Fall diet						
Species, Red fox	− 0.21 (0.22)	1	0.339	− 0.01 (0.30)	1	0.981
Sex, Male	− 0.55 (0.16)	1	< 0.001*	− 0.26 (0.21)	1	0.228
Region, Ungava	0.22 (0.23)	1	0.346	0.24 (0.28)	1	0.389
Status of infection	0.63 (0.18)	1	< 0.001*	0.68 (0.24)	1	0.004*
Winter diet						
Species, Red fox	− 0.22 (0.18)	1	0.243	− 0.03 (0.23)	1	0.896
Sex, Male	− 0.28 (0.14)	1	0.044*	− 0.22 (0.18)	1	0.219
Region, Ungava	− 0.36 (0.20)	1	0.081	− 0.21 (0.22)	1	0.333
Status of infection	0.55 (0.16)	1	< 0.001*	0.43 (0.19)	1	0.025*

β, estimate coefficient; SE, standard error; *df*, degree of freedom.

*Statistically significant at p < 0.05.

#### Logistic regression: fall diet

When fox species were combined, foxes consuming migratory geese or cached eggs in fall were 2 times more likely to be positive for *T. gondii* (odds ratio: 1.86, CI_95%_:1.17–2.98, p = 0.009, Table 4). For red foxes, the odds of being positive for *T. gondii* were 3 times higher in foxes consuming aquatic food sources (odds ratio: 2.95, CI_95%_:1.43–6.05, p = 0.003, Table 4).

**Table 4:** Coefficients of the final logistic regression model and risk factors associated with *Toxoplasma gondii* prevalence in foxes (*Vulpes* spp.) from Nunavik, QC.

Variables fall diet	β (SE)	p-value	df	OR (CI_95%_)
Species, Red fox	3.81 (2.80)	0.174	1	44.96 (0.19–1.08e + 04)
Region, Ungava	− 1.59 (1.30)	0.223	1	0.20 (0.02–2.62)
Dietaquatic	− 0.82 (0.33)	0.014*	1	0.44 (0.23–0.85)
Dietmigration	0.62 (0.24)	0.009*	1	1.86 (1.17–2.98)
Species^Region, Red fox Ungava	− 0.09 (1.40)	0.950	1	0.92 (0.06–14.33)
Species^Dietaquatic, Red fox	1.08 (0.37)	0.003*	1	2.95 (1.43–6.05)
Species^Dietmigration, Red fox	− 0.64 (0.25)	0.009*	1	0.53 (0.32–0.85)

Variables winter diet				
Species, Red fox	5.71 (3.47)	0.100	1	303.29 (0.34–2.74e + 05)
Region, Ungava	0.64 (0.91)	0.484	1	1.89 (0.32–11.26)
Dietaquatic	− 0.05 (0.05)	0.308	1	0.95 (0.86–1.05)
Dietmigration	0.24 (0.11)	0.030*	1	1.27 (1.02–1.58)
Species^Region, Red fox Ungava	− 1.45 (1.00)	0.148	1	0.23 (0.03–1.67)
Species^Dietaquatic, Red fox	0.26 (0.12)	0.029*	1	1.29 (1.03–1.63)
Species^Dietmigration, Red fox	− 0.24 (0.12)	0.050*	1	0.78 (0.61–1.00)

*^* , interaction term; β, estimate coefficient; SE, standard error; *df*, degree of freedom; OR, odds ratio; CI_95%_, 95% confidence interval.

*Statistically significant at p < 0.05.

#### Logistic regression: winter diet

When fox species were combined, foxes consuming migratory geese (most likely from the contribution of cached eggs) in winter were 1.3 times more likely to be positive for *T. gondii* (odds ratio: 1.27, CI_95%_:1.02–1.58, p = 0.030, Table 4). As well, red foxes consuming aquatic food sources were 1.3 times more likely to be exposed to *T. gondii* (odds ratio: 1.29, CI_95%_:1.03–1.63, p = 0.029, Table 4). The relationship between consuming geese and *T. gondii* infection was stronger for Arctic foxes in both fall and winter diet (Table 4).

## Discussion

This study combines ecological trophic analysis (through stable isotope signatures) with an epidemiological study of infection status of a food borne pathogen in terrestrial mammals, demonstrating that red and Arctic foxes positive for *T. gondii* were more likely to consume aquatic prey and migratory geese, respectively, and not rodents, their major food source^29^. Arctic and red foxes were previously hypothesized to be good sentinels for *T. gondii* circulation in northern ecosystems as they are widespread across northern Canada, exposed at similar rates and routes as humans in many northern regions, and can be exposed to both oocysts shed into the environment by felids and tissue cysts in consumed prey^11,13^. The likelihood of a fox being positive for *T. gondii* increased significantly with stable isotope values of δ^15^N (Table 3). This suggests that positive foxes feed at higher trophic levels, and/or on prey with high values of δ^15^N that are more likely to be infected with the parasite (i.e., other carnivores). This is consistent with transmission through carnivory or scavenging, supporting our hypothesis that this is a major route of exposure for foxes versus direct oocyst transmission from the environment. Since lemmings are year round resident herbivores (Centre d’études nordiques, unpublished data) in a region where felids are largely absent and less likely to contaminate the environment with oocysts, exposure through this particular prey is less likely; *T. gondii* infection in rodents has not been reported in Arctic tundra ecosystems^5,9^.

Although ultimately of terrestrial origin, *T. gondii* can bioaccumulate in aquatic organisms, playing a significant role in foodborne and waterborne transmission^48^—a “pathogen pollutant” in aquatic ecosystems. Sporulated oocysts persist in aquatic environments and are resistant to temperature variations^49^. Red foxes consuming aquatic food sources in fall and winter were more likely to be exposed to the parasite. Spillover to terrestrial wildlife and humans through consumption of aquatic wildlife in the Arctic is often regarded as a possible hypothesis for transmission^3,4,50^. Coastal areas, in particular, often receive substantial inputs of energy and nutrients from the ocean, and these resources can support large numbers of consumers^17^. Roth et al.^14^ found that the stable-carbon isotope ratios of Arctic fox hair indicated the diet was much more marine in winter, probably due to increased access to marine food sources such as carcasses of seals killed by polar bears and/or hunters via sea ice. Killengreen et al.^51^ also found that red foxes close to the coast in winter had strong isotopic signatures of marine components, and rely on these resources when terrestrial prey became scarce. We found that female foxes had a higher input of δ^13^C, indicating that female foxes consumed more marine food sources. This may be due to higher metabolic demands for female foxes raising pups, resulting in females staying closer to coastal areas than males, who spend more time dispersing and moving between territories^52^. Although foxes are known to travel long distances for breeding and dispersal, foxes tend to settle in high-quality habitat patches where prey density is high, and often close to their natal areas^53^. For this reason, we are relatively confident that foxes sampled in the study were consuming local prey in the months prior to being trapped; i.e., given our wide study area, most juvenile foxes that we sampled would be undergoing natal dispersal^54^ rather than migrating from outside Nunavik.

Foxes consuming migratory geese for fall diet were twice as likely to be positive for *T. gondii*, especially Arctic foxes. This is not unexpected given previous epidemiological studies showing that migratory geese (mostly *Branta canadensis*) in Nunavik harbor *T. gondii*, that Inuit consuming waterfowl are at higher risk of being exposed to the parasite, and that migratory birds are a likely source of *T. gondii* exposure for foxes^3,25^. Foxes in our study area would have access to nesting geese and their eggs, and migratory geese passing through on their way south from May until late September. The highest densities of breeding Canada geese are found in the two main regions of Nunavik where foxes were trapped: coastal lowlands of eastern Hudson Bay and coastal lowlands of southwestern Ungava Bay^55^. Although Arctic foxes are thought to rely heavily on fluctuating rodent populations, having a regular large influx of birds (and cached eggs) provides foxes with predictable food resources; Arctic foxes are well-known to exhibit hoarding behavior and cached eggs can be heavily utilized during fall and winter months^56–58^. This behavior could explain the higher prevalence of *T. gondii* in Arctic vs red foxes in our study (Table 2), with greater reliance on these migratory geese.

We observed higher sero- and tissue prevalence in foxes from Hudson Bay (65% and 51%, respectively) compared to Ungava Bay (29% and 20%, respectively). A similar pattern is also seen in people, with a seroprevalence of 56% in Hudson Bay and 37% in Ungava Bay^3^. In people, this was explained by a higher consumption of marine mammals, fish, and geese in Hudson Bay and Hudson Strait compared to Ungava Bay^3,59,60^. We hypothesize that the same scenario could be happening in foxes, with those in Hudson Bay having greater access to migratory birds, fish, and carcasses of marine mammals, which could be an important source of *T. gondii*. As well, contamination of fishes and marine mammals could be related to the geography of the watersheds that irrigate Nunavik. The Hudson watershed originates mostly from subarctic and boreal regions, where lynx could be shedding oocysts in the environment, while the Ungava watershed is restricted to the tundra for the most part^3,50,61^.

Epidemiological study limitations include disagreement between detection methods for *T. gondii:* 26 foxes were positive on serology, but DNA was not detected in tissues. This discrepancy is expected and is frequently explained by acute exposure where tissue invasion has not yet occurred, low tissue infection intensity (below the detection limit of the molecular technique), and/or non-uniform distribution of tissue cysts of *T. gondii*^11^. As well, false-seropositive results could occur due to the high blood content in heart juice which may have interfered with antibody binding^62^, or from cross-contamination between samples (less likely since negative controls remained negative). Only one fox was positive for DNA in tissues and negative on serology. This individual could be acutely infected and had not yet developed antibodies, or harbor a senescent infection i.e., antibodies against *T. gondii* have declined, with tissue cysts persisting in a non-immunogenic state^38,63^. We acknowledge sampling bias in estimating prevalence using trapped foxes; infection with *T. gondii* has been linked to increased risk behaviours, such as entering a trap^64^. Therefore, the proportion of positive animals in this study may overestimate the prevalence of toxoplasmosis in the population, but also ensured that we had an adequate sample size of positive foxes to explore dietary links. Limitations of the trophic ecological aspects of the study include our inability to dictate time of year of fox trapping by community collaborators (fur harvest occurs only in winter), migratory and other movements of wildlife, and the use of stable isotope analysis as an indirect method for determining animal diets. As there is no a priori information available for the diet of foxes in the present study, we exercise caution when interpreting the results since there can be considerable inter- and intra-population variability in diet^65^. We also acknowledge that we did not sample all possible food sources in our choice of dietary endpoints (e.g., scavenging on caribou carcasses), but instead focused on three most relevant preys for foxes at the local scale.

## Conclusion

Our findings support that stable isotope analysis is a powerful tool for tracking food-borne parasite transmission through food webs. We worked with community harvesters and combined epidemiological and ecological methods to unravel the complex transmission of a ubiquitous food-borne pathogen (*T. gondii*) in a remote environment. While parasites and trophic relationships have been studied before^66,67^, very few have linked infection status with trophic relationships using stable isotopes^68^. Our results suggest that non-invasive hair sampling coupled with serology holds promise for in vivo approaches to source attribution of food-borne pathogens, especially in wildlife of conservation significance, and in people. We found that migratory geese and aquatic food sources are potentially a significant contributor to the transmission of *T.* gondii in wildlife sentinels in northern regions, echoing recent surveys in people in Nunavik^3^. Such baseline data has never been more important, as the Arctic is experiencing unprecedented temperature and precipitation change^69^. With increased climate variability and extreme weather events, the ecology and transmission of *T. gondii* is expected to shift^70^. The melting of ice and permafrost, in addition to increased precipitation, will likely boost the transport of oocysts in northern ecosystems. As well, warmer temperatures will favor oocyst survival and development^71^. As the tree line moves northward, the habitat range of lynx and their prey species will likely follow, also possibly affecting fox and human diets in northern ecosystems^72^. Understanding current trophic relationships and parasite transmission in foxes as a sentinel system will allow us to detect and predict changes in a rapidly warming Arctic, including altered zoonotic risk for northern human populations in the Arctic, who remain intricately linked to wildlife populations and the land.

### Supplementary Information

Supplementary Information.

## Data Availability

All data relating to this study are available on request from the corresponding author.
