# Supplementary material for: Use of stable isotopes to reveal trophic relationships and transmission of a food-borne pathogen
Source: Sci Rep. 2024 Feb 2;14:2812. doi: 10.1038/s41598-024-53369-6 (PMC10837197; doi:10.1038/s41598-024-53369-6)
Supplement: Supplementary file 1 — Supplementary Information. [file 41598_2024_53369_MOESM1_ESM.docx]

**Use of stable isotopes to reveal trophic relationships and transmission of a food-borne pathogen**

Émilie Bouchard, Michaël Bonin, Rajnish Sharma, Adrián Hernández-Ortiz, Géraldine-G. Gouin, Audrey Simon, Patrick Leighton & Emily Jenkins

**Supplementary material**

**Table S1.** Carbon (δ^13^C) and nitrogen (δ^15^N) isotopic ratios (‰, mean ± SD) of food sources used in stable isotope analyses of foxes with corresponding tissue types and sample sizes.

| **Food sources** | **Dietary endpoints** | **δ^13^C, ‰** | **δ^15^N, ‰** | **Tissue type** | **Sample size** |
| --- | --- | --- | --- | --- | --- |
| Lemming | Terrestrial | -25.52 ± 0.7 | 2.76 ± 1.5 | Hair | 3 |
| Goose | Migratory | -22.53 ± 1.3 | 6.45 ± 1.0 | Muscle | 26 |
| Fish | Aquatic | -18.2 ± 1.5 | 14.71 ± 0.9 | Muscle | 13 |

**Table S2. A)** Summary of seasonal stable isotope analyses between regions in both species of foxes from Nunavik, QC. Results are given as mean proportion of dietary endpoints in the diet of foxes at the population scale.

|  | **Fall diet** | | | | **Winter diet** | | | |
| --- | --- | --- | --- | --- | --- | --- | --- | --- |
| Dietary endpoints | **Red fox** | | **Arctic fox** | | **Red fox** | | **Arctic fox** | |
|  | x̄ | CI_95%_ | x̄ | CI_95%_ | x̄ | CI_95%_ | x̄ | CI_95%_ |
| **Ungava Bay** | n=99 | | n=12 | | n=143 | | n=19 | |
| Terrestrial | 88 | (82-91) | 82 | (66-93) | 86 | (81-90) | 67 | (43-82) |
| Migratory | 4 | (1-11) | 11 | (2-30) | 6 | (1-14) | 20 | (3-52) |
| Aquatic | 8 | (5-10) | 6 | (1-13) | 8 | (5-10) | 13 | (3-22) |
| **Hudson Bay** | n=30 | | n=12 | | n=38 | | n=21 | |
| Terrestrial | 84 | (73-92) | 75 | (58-87) | 77 | (63-86) | 56 | (36-70) |
| Migratory | 9 | (1-23) | 16 | (2-37) | 14 | (2-15) | 22 | (3-52) |
| Aquatic | 7 | (2-12) | 9 | (2-16) | 9 | (3-15) | 22 | (11-30) |

**Table S2. B)** Summary of seasonal stable isotope analyses between *Toxoplasma gondii* exposure status in both species of foxes from Nunavik, QC. Results are given as mean proportion of dietary endpoints in the diet of foxes at the population scale.

|  | **Fall diet** | | | | | | **Winter diet** | | | |  |
| --- | --- | --- | --- | --- | --- | --- | --- | --- | --- | --- | --- |
| Dietary endpoints | **Red fox** | | | **Arctic fox** | | | **Red fox** | | **Arctic fox** | |  |
|  | x̄ | CI_95%_ | | x̄ | | CI_95%_ | x̄ | CI_95%_ | x̄ | CI_95%_ |  |
| **Positive foxes** | n=50 | | n=11 | | | | n=69 | | n=19 | | |
| Terrestrial | 82 | (72-88) | 72 | | (55-85) | | 76 | (64-84) | 56 | (35-70) | |
| Migratory | 8 | (1-23) | 18 | | (3-40) | | 13 | (2-33) | 24 | (3-54) | |
| Aquatic | 10 | (5-15) | 9 | | (2-17) | | 10 | (4-15) | 20 | (10-29) | |
| **Negative foxes** | n=79 | | n=13 | | | | n=112 | | n=21 | | |
| Terrestrial | 91 | (85-95) | 82 | | (67-93) | | 89 | (84-92) | 64 | (41-80) | |
| Migratory | 4 | (1-11) | 12 | | (2-29) | | 5 | (1-13) | 22 | (3-54) | |
| Aquatic | 5 | (2-8) | 6 | | (1-13) | | 6 | (3-8) | 14 | (4-24) | |

**
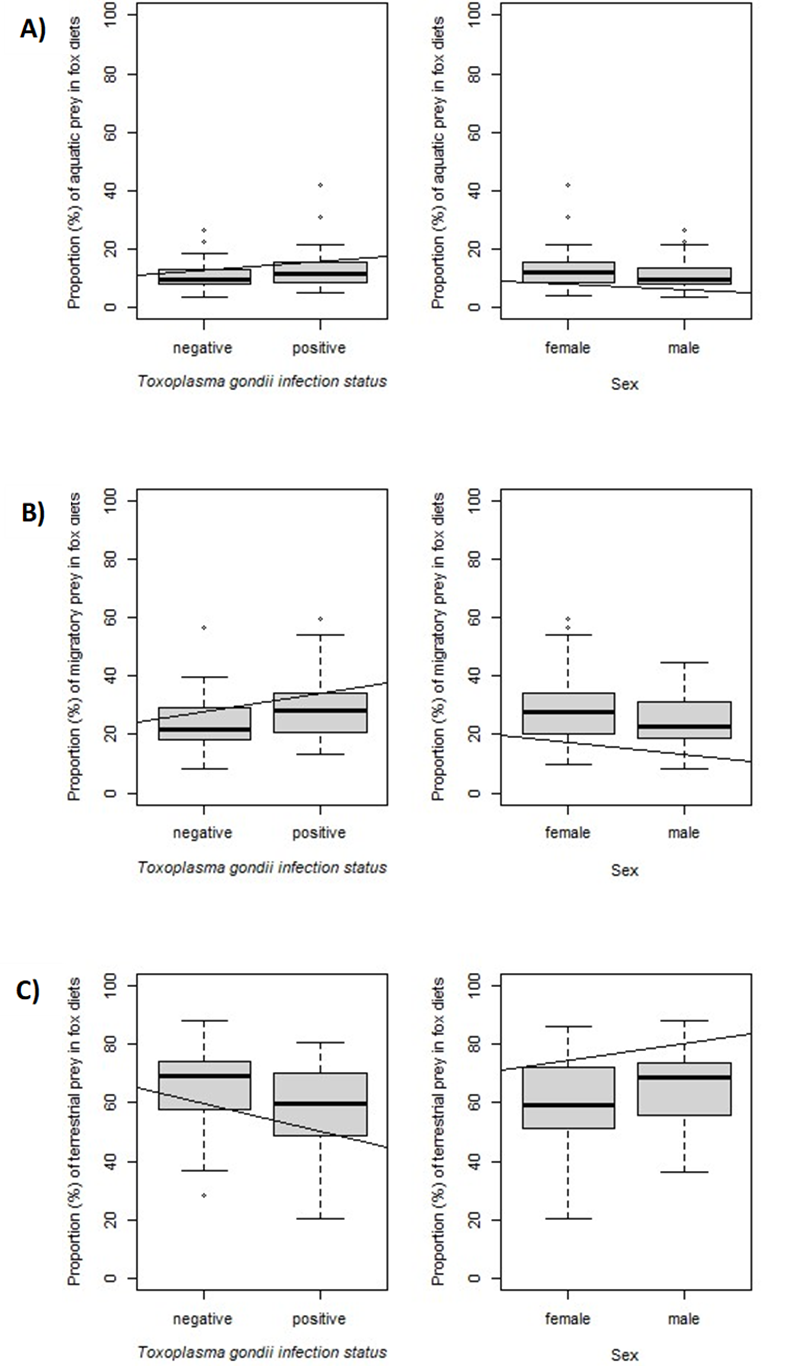
**

**Figure S1.** Proportion values of each dietary endpoints (aquatic prey (A), migratory prey (B), and terrestrial prey (C)) to the diet of foxes with status of infection or sex as response variables.
